# Alcohol and Puberty: Mechanisms of Delayed Development

**DOI:** 10.35946/arcr.v38.2.09

**Published:** 2017

**Authors:** William L. Dees, Jill K. Hiney, Vinod K. Srivastava

**Affiliations:** William L. Dees, Ph.D., is a Professor; Jill K. Hiney, Ph.D., is a Research Assistant Professor; and Vinod K. Srivastava, Ph.D., is a Research Associate Professor, all in the Department of Veterinary Integrative Sciences, College of Veterinary Medicine, Texas A&M University, College Station, Texas

**Keywords:** Alcohol consumption, alcohol use and misuse, adverse effects, adolescence, puberty, development, brain, hypothalamus, hypothalamic function, neuroendocrine system

## Abstract

Adolescence represents a vulnerable period for developing youth. Alcohol use and misuse are especially problematic behaviors during this time. Adolescents are more sensitive to alcohol and less tolerant of its detrimental effects than are adults. Research in humans and animals has revealed that early alcohol consumption can result in delayed pubertal development. Animal studies have shown that alcohol detrimentally affects neuroendocrine systems within the hypothalamic region of the brain that are associated with the normal, timely onset of the pubertal process. To effectively restore development and shorten recovery time associated with the adverse effects of alcohol on puberty, researchers must first understand the molecular and physiological mechanisms by which alcohol interferes with critical hypothalamic functions.

Despite efforts to prevent underage alcohol use, drinking does occur as early as the 6th grade. According to a recent national survey, 9.7 percent of 8th graders and 21.5 percent of 10th graders reported using alcohol at least once in the previous 30 days (Johnston et al. 2016). This is important because people who begin drinking between ages 11 and 14 are at increased risk for developing alcohol use disorder (DeWit et al. 2000), compared with those who begin drinking at later ages. These high-risk age groups also are exactly within the pubertal time frame. Some of the younger adolescents may not have begun the pubertal process. Others, however, are subject to the process being slowed or halted by alcohol, thus impeding further development. Following a brief summary of alcohol’s effects on puberty in humans, this review describes the neuroendocrine processes that control puberty and research using animal models to assess the effects of prepubertal alcohol exposure.

Early research demonstrated that alcohol use by adolescent boys causes suppressed serum levels of growth hormone (GH), luteinizing hormone (LH), and testosterone (Diamond et al. 1986; Frias et al. 2000*a*,*b*), as well as lower bone density (Fehily et al. 1992; Neville et al. 2002). In adolescent girls, alcohol use caused suppressed serum GH and estradiol (E_2_) levels (Block et al. 1993; Frias et al. 2000*b*). Other studies found evidence for disruptions in stature, weight distribution, and a risk for nutritional deficiencies (Block et al. 1991; Yamamoto et al. 1991). More recently, studies in girls have shown that prepubertal alcohol use was associated with delayed breast development (Peck et al. 2011) and onset of menarche (Richards et al. 2011). This research suggested that prepubertal girls who use alcohol have four times the chance of delayed onset of puberty than those who do not (Peck et al. 2011). This finding is confirmed in animal models, which show that alcohol acts within the hypothalamic region of the brain to suppress key puberty-related genes and hormones responsible for the normal timing of development.

## Basic Neuroendocrine Control of Puberty

The onset of puberty results from a complex series of interactions between nerve cells (i.e., neurons) and glial cells (i.e., nonneuronal brain cells) within the hypothalamus that are governed by metabolic signals, as well as genetic and environmental influences. Although age at puberty varies widely between and among mammalian species, the main event that signals puberty onset is basically similar, in that it relies on the increased pulsatile secretory activity of a hypothalamic neuropeptide, luteinizing hormone–releasing hormone (LHRH). This event occurs through the enhanced developmental responsiveness of the LHRH-producing neurons and their nerve terminals to excitatory inputs, such as insulin-like growth factor-1 (IGF-1) (Hiney et al. 1996; Wilson 1998) and the kisspeptins (Kp), a family of neuropeptide products of the *KiSS-1* gene (Navarro et al. 2004; Shahab et al. 2005), as well as leptin (Dearth et al. 2000; Lebrethon et al. 2000), transforming growth factor α (Ojeda et al. 1990), and excitatory amino acids (Claypool et al. 2000; Gay and Plant 1987; Urbanski and Ojeda 1990).

In addition to the development of excitatory inputs, the timing of puberty is influenced by a concomitant and gradual removal of prepubertal inhibitory inputs*,* such as γ aminobutyric acid (GABA) and the opioid peptides β endorphin and dynorphin (Lehman et al. 2010; Navarro et al. 2009; Srivastava et al. 2015; Terasawa and Fernandez 2001). This alteration, often referred to as a “brake” on the pubertal process, is responsible for keeping prepubertal LHRH secretion low. As LHRH secretion increases, it drives the timing of puberty in both sexes by stimulating pituitary gonadotropin secretions, which in turn stimulate gonadal steroid synthesis and secretions for further maturation of the hypothalamus and reproductive organs. Although all of the excitatory and inhibitory influences noted above have been shown to be involved in the pubertal process, the mechanism-of-action portion of this review will concentrate on the most current findings about some of these modulators in relation to their upstream and downstream influences on the pubertal process.

## Overall Effects of Alcohol on Puberty-Related Hormones and Indices of Pubertal Development

Initial studies using both female and male rodents revealed that chronic alcohol administration caused delayed puberty (Anderson et al. 1987; Bo et al. 1982; Ramaley 1982). Over the years, researchers have attempted to correlate the timing of puberty with specific puberty-related hormones following chronic prepubertal alcohol exposure. In female rats, alcohol caused delayed vaginal opening and the age at first estrus (Dees and Skelley 1990; Emanuele et al. 2002), as well as suppressed serum levels of GH and LH but not follicle-stimulating hormone (FSH) (Dees and Skelley 1990). In this regard, the differential effects of alcohol on LH and FSH were not surprising, because this previously had been shown in adult rats (Dees and Kozlowski 1984). Significantly, several studies have shown that prepubertal alcohol exposure in females caused suppressed circulating levels of E_2_ (Bo et al. 1982; Dees and Skelley 1990; Emanuele et al. 2002), a clear indication of impaired ovarian development and activity. Although less is known about the prepubertal effects of alcohol in males, it has been shown to cause an early suppression in serum LH (Cicero et al. 1990) and to reduce the serum levels of GH and testosterone. Prepubertal alcohol use also can lead to lower testicular weight and smaller secondary sex organs (Anderson et al. 1987; Cicero et al. 1990; Emanuele et al. 1999; Tentler et al. 1997).

Additional research conducted in an animal model that more closely resembled humans, female rhesus monkeys, found that chronically administered alcohol resulted in suppressed GH, LH, and E_2_ (Dees et al. 2000), exactly as described above in immature female rats. Furthermore, these actions were associated with the altered development of a regular monthly pattern of menstruation (Dees et al. 2000).

In addition to the effects of alcohol on GH and LH, research has shown that prepubertal alcohol administration caused suppressed serum IGF-1 in immature female rats (Emanuele et al. 2002; Srivastava et al. 1995) and rhesus monkeys (Dees et al. 2000), thereby reducing the amount of peptide available to the prepubertal hypothalamus. This is relevant because IGF-1 normally can act centrally to influence both the hypothalamic–pituitary–gonadal axis and the hypothalamic–pituitary GH axis at puberty. Specifically, IGF-1 has been shown to act at the hypothalamic level to stimulate LHRH/LH secretion (Hiney et al. 1991, 1996) and advance the time of puberty in female rodents (Danilovich et al. 1999; Hiney et al. 1996). The ability of IGF-1 to regulate GH through its actions on hypothalamic growth hormone–releasing hormone and somatostatin (i.e., somatotropin release–inhibiting factor), the latter being a GH-release inhibitor, have been well documented (for review, see Bercu 1996).

It is important to note that the central control of these two hypothalamic systems is complex and interrelated, especially regarding the important integrative and bidirectional influences of IGF-1 on their respective neuro-secretions. Although a detailed discussion of these basic interrelationships is beyond the scope of this review, it also is worth noting that alcohol can affect both of these systems at multiple levels. For example, in addition to the aforementioned alcohol-related suppression of LHRH/LH resulting in suppressed serum E_2_, alcohol also causes altered hypothalamic growth hormone–releasing hormone synthesis and secretion (Dees et al. 1990). This then results in decreased pulsatile GH release (Dees et al. 1988), which in turn downregulates IGF-1 synthesis by liver hepatocytes (Srivastava et al. 2002). The resulting alcohol-induced suppression in circulating IGF-1 (Srivastava et al. 1995) causes suppressed body growth and interferes with the maturation and function of several organ systems. Furthermore, the accompanying reduction in circulating IGF-1 to feedback on the hypothalamus further reduces the secretion of LH and GH (for review, see Dees et al. 2009).

All of the above hormones are critical for puberty. However, alcohol’s suppression of the pituitary secretion of LH has become a primary focus of research on pubertal onset, because this gonadotropin is regulated by LHRH, the hypothalamic peptide responsible for beginning the pubertal process. Researchers now are examining whether the alcohol-induced effect to suppress LH is a result of a hypothalamic or pituitary site of action.

## The Hypothalamic Site of Alcohol’s Actions

Studies in female rats, which showed increased hypothalamic LHRH content after chronic prepubertal alcohol administration (Dees et al. 1990), offered the first indirect evidence that alcohol affects this part of the brain. Subsequently, alcohol was shown to block the stimulatory effects of norepinephrine (Hiney and Dees 1991), IGF-1 (Hiney et al. 1998), leptin (Hiney et al. 1999), and *N*-methyl-dl-aspartic acid (NMA) (Nyberg et al. 1993) on the in vitro release of prepubertal LHRH. Although important, these collective observations did not rule out the possibility that alcohol also may act at the level of the pituitary.

To definitively assess the site of alcohol action, prepubertal rhesus monkeys that had been chronically exposed to alcohol were subjected to hypothalamic and pituitary response tests (Dissen et al. 2004). The hypothalamic stimulation test showed that the NMA-induced LH secretion observed in the non–alcohol-treated monkeys was blocked in the alcohol-treated monkeys. This is significant, because NMA causes LH release by first stimulating hypothalamic LHRH secretion and does not act at the pituitary level. Three weeks later, these same animals were given LHRH to test pituitary responsiveness. Results indicated that the LH response to the peptide was the same in both non–alcohol-treated and alcohol-treated monkeys, conclusively demonstrating the hypothalamic site of action.

## Mechanisms of Action

### Upstream Effects of Alcohol on LHRH Synthesis

**Figure 1:**
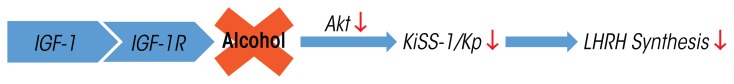
Alcohol blocks the ability of insulin-like growth factor-1 (IGF-1) to induce the *KiSS-1* gene and therefore suppresses production of kisspeptins (Kp), a family of neuropeptide products of *KiSS-1*, by inhibiting IGF-1 receptor (IGF-1R)-induced phosphorylation of Akt, a transduction signal that mediates the actions of IGF-1. Suppressed Kp production subsequently results in reduced synthesis of luteinizing hormone–releasing hormone (LHRH). SOURCE: Hiney et al. 2010.

The majority of LHRH-synthesizing neurons are localized within the brain preoptic area and the region just posterior to it referred to as the anterior hypothalamic area. This latter area also contains the anteroventral periventricular (AVPV) nucleus. Neurons in the AVPV nucleus produce kisspeptins, which regulate prepubertal LHRH synthesis and are critical for the onset of puberty (de Roux et al. 2003; Keen et al. 2008; Navarro et al. 2004; Shahab et al. 2005). Thus, research focused on discerning which factors affect prepubertal *KiSS-1* expression. Chronic prepubertal alcohol exposure was shown to cause suppressed *KiSS-1* gene expression in the AVPV nucleus of female rats, an action associated with a decrease in the usual level of phosphorylated Akt (Srivastava et al. 2009). Akt is a transduction signal that mediates the actions of IGF-1 (Cardona-Gomez et al. 2002), a peptide known to activate puberty in rats and rhesus monkeys (Hiney et al. 1996; Wilson 1998). Understanding IGF-1’s ability to regulate *KiSS-1* was essential to further research. In studies with rats, an injection of IGF-1 directly into the brain’s third ventricle caused the upregulation of prepubertal *KiSS-1* gene expression in the AVPV nucleus 6 hours later (Hiney et al. 2009). Subsequently, alcohol was shown to block the IGF-1 induction of *KiSS-1* in the AVPV nucleus by inhibiting IGF-1 receptor (IGF-1R)-induced phosphorylation of Akt (Hiney et al. 2010). Figure 1 depicts this alcohol action, which leads to suppressed Kp and, subsequently, suppression of LHRH synthesis.

Further investigation will determine whether the suppressed Akt activity occurred directly at the level of Kp-containing neurons or through an interneuron or glial cell that also expresses the IGF-1R. However, the fact that alcohol can interfere with this pathway to LHRH synthesis is important, because once the onset of puberty begins, the synthesis of this peptide must keep pace with its release to drive the pubertal process.

### Downstream Effects of Alcohol on LHRH Release

Alcohol is known to alter several downstream signals in the hypothalamus that collectively reduce LHRH release at puberty. Although the numerous excitatory substances mentioned above influence LHRH at puberty, the role of *KiSS-1* and Kp also are noteworthy. *KiSS-1* expression increases in the hypothalamus as puberty approaches (Navarro et al. 2004), and Kp is a potent stimulator of prepubertal LHRH secretion (Keen et al. 2008; Navarro et al. 2004). By suppressing prepubertal *KiSS-1*/Kp (Srivastava et al. 2009), alcohol contributes to decreased LHRH secretion at a time when increases are needed as puberty approaches. In addition, alcohol has been shown to stimulate the release of GABA and the opioid peptides (Lomniczi et al. 2000), which, as stated above, are known inhibitors of LHRH release. Alcohol also can activate the hypothalamic–pituitary–adrenal axis (Rivier 1996), and the hormones involved in the stimulation of this stress axis can suppress LH secretion (Kinsey-Jones et al. 2009; Li et al. 2015). Furthermore, the newly described gene *Lin28b* also is associated with the brake on puberty, and its expression has been shown to gradually decrease as puberty approaches (Sangiao-Alvarellos et al. 2013).

**Figure 2:**
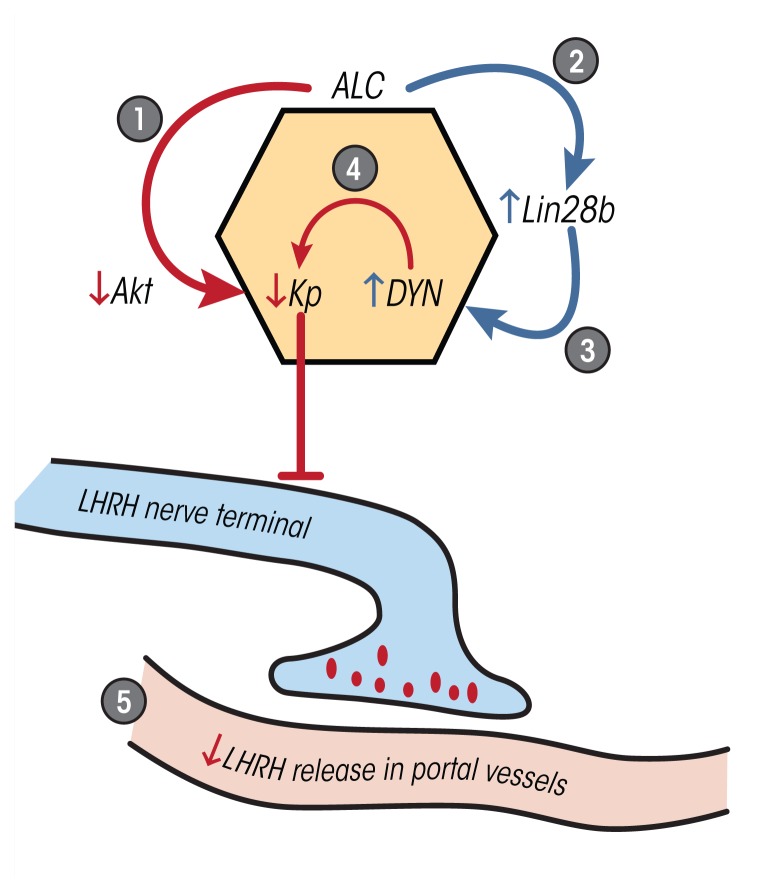
Schematic showing the effects of alcohol (ALC) on critical pathways within the hypothalamus that contribute to the control of luteinizing hormone–releasing hormone (LHRH) secretion. **(1)** Alcohol inhibits Akt, a transduction signal that mediates the actions of insulin-like growth factor-1 (IGF-1). This results in suppressed synthesis of kisspeptins (Kp), peptides that stimulate LHRH secretion. **(2)** Alcohol prevents the normal pubertal decline in the expression of Lin28b, a gene associated with the brake on puberty, by stimulating its synthesis. **(3)** Lin28b then stimulates synthesis of dynorphin (DYN), a peptide that inhibits Kp. **(4)** Alcohol stimulates the release of inhibitory DYN to suppress Kp. **(5)** The suppressed Kp ultimately results in decreased LHRH release. Red indicates suppression/inhibition; Blue indicates stimulation. For clarity, other factors contributing to LHRH release are not shown.

Recent research assessed whether alcohol would alter the normal pubertal rise in Kp and decrease in Lin28b protein. Chronic alcohol exposure reversed these actions within the brain region known as the medial basal hypothalamus (MBH) in prepubertal female rats by suppressing Akt, *KiSS-1*, and Kp (Srivastava et al. 2009, 2015), while stimulating the synthesis of Lin28b (Srivastava et al. 2015). In addition, research showed that Lin28b induced dynorphin (DYN) synthesis and that alcohol stimulated DYN release (Srivastava et al. 2015). DYN inhibits Kp and LHRH secretion (Lehman et al. 2010; Navarro et al. 2009). Because the MBH contains neurons that coexpress Kp and DYN, these observations are relevant to the control of prepubertal LHRH secretion. Figure 2 illustrates the simultaneous and differential effects of alcohol on the excitatory Kp and inhibitory Lin28b pathways. Although LHRH neurons are not localized within the MBH of the rat, they are in primates, including humans. Therefore, both the release and synthesis of LHRH in the MBH of primates may be affected by alcohol.

In addition to alcohol’s actions on neuronal inputs controlling prepubertal LHRH secretion discussed above, alcohol may affect neuronal-to-glial and glial-to-glial inputs facilitating LHRH release within the MBH. LHRH secretory activity can be modulated by a specific neuronal-glial gene family that synthesizes signaling proteins involved in bidirectional communications at puberty (Ojeda et al. 2010). Chronic prepubertal alcohol exposure decreases the synthesis of glial protein tyrosine phosphatase-β, which is required for binding to the neuronal components contactin and contactin-associated protein-1. This finding demonstrates that alcohol can alter these interactions and interfere with glial–neuronal communications (Srivastava et al. 2011*a*).

Glial-to-glial interactions also are affected by alcohol. Once released, glial-derived epidermal growth factor and transforming growth factor α (TGFα) both bind to the erbB1 receptor on adjacent glial cells and stimulate the release of prostaglandin E_2_ (PGE_2_) (Ma et al. 1997), a well-known stimulator of LHRH secretion. Alcohol exposure initially was shown to inhibit PGE_2_ release induced by epidermal growth factor/TGFα (Hiney et al. 2003). In addition, glial-derived IGF-1 binds to IGF-1R on adjacent glial cells, which produce TGFα, and alcohol exposure altered the synthesis and release of TGFα (Srivastava et al. 2011*b*) and PGE_2_ (Hiney et al. 1998; Srivastava et al. 2011*b*), thereby resulting in decreased prepubertal LHRH secretion. Furthermore, specialized glial cells within the MBH known as tanycytes release glial-derived TGFβ1, causing retraction of their processes and allowing for better entry of LHRH into the system of blood vessels that connect the hypothalamus with the pituitary (i.e., hypophyseal portal system) (Prevot et al. 2003). Alcohol blocks IGF-1 from stimulating the synthesis and release of TGFβ1 by altering the IGF-1R synthesis and Akt phosphorylation, therefore further contributing to diminished LHRH secretion (Hiney et al. 2014).

## Conclusion

Alcohol use and misuse by adolescents increases the risk for altered neuro-endocrine function, potentially modifying the timing of pubertal development. This review highlights results of research with animal models showing the site and mechanisms by which alcohol causes puberty-related problems. These studies demonstrate that alcohol acts within the hypothalamus to alter the expression and function of excitatory and inhibitory puberty-related genes and neuro-hormones, which are critical for the timely increase in LHRH secretion and the onset of puberty. More research in this field is needed and would no doubt promote a better understanding of normal mechanisms controlling events leading to increased LHRH release at puberty, as well as the cause-and-effect relationships by which alcohol can differentially affect them.

Advancing knowledge in this area will allow researchers to begin to identify potential treatment substances that may lessen the impact and shorten the recovery time of adolescents who show signs of delayed development associated with alcohol use and misuse. It also is significant that delayed puberty is known to be associated with altered gonadal steroid production, which is needed for the development and function of several body systems. Furthermore, delayed pubertal development correlates with other health concerns such as altered bone density or height and weight issues, as well as psychological problems. Thus, the neuroendocrine consequences of alcohol use can result in far-reaching adolescent health concerns.
